# Unveiling personalized and gamification-based cybersecurity risks within financial institutions

**DOI:** 10.7717/peerj-cs.2598

**Published:** 2025-02-07

**Authors:** Amna Shahzadi, Kashif Ishaq, Naeem A. Nawaz, Fadhilah Rosdi, Fawad Ali Khan

**Affiliations:** 1 School of Systems and Technology, University of Management and Technology, Lahore, Pakistan; 2 Faculty of Information Science and Technology, Universiti Kebangsaan Malaysia, Bangi, Malaysia

**Keywords:** Gamification, Cyber security, Prevention measures, Artificial intelligence, User behavior, Financial sector

## Abstract

Gamification has emerged as a transformative e-business strategy, introducing innovative methods to engage customers and drive sales. This article explores the integration of game design principles into business contexts, termed “gamification,” a subject of increasing interest among both scholars and industry professionals. The discussion systematically addresses key themes, like the role of gamification in marketing strategies, enhancing website functionality, and its application within the financial sector, including e-banking, drawing insights from academic and industry perspectives. By conducting a systematic literature review of 48 academic articles published between 2015 and 2024, this study examines the use of personalized, gamification-based strategies to mitigate cyber threats in the financial domain. The review highlights the growing digitization of financial services and the corresponding rise in sophisticated cyber threats, including traditional attacks and advanced persistent threats (APTs). This article critically assesses the evolving landscape of cyber threats specific to the financial industry, identifying trends, challenges, and innovative solutions to strengthen cybersecurity practices. Of particular interest is the application of AI-enhanced gamification strategies to reinforce cybersecurity protocols, particularly in the face of novel threats in gaming platforms. Furthermore, the review evaluates techniques grounded in user behavior, motivation, and readiness to enhance cybersecurity. The article also offers a comprehensive taxonomy of financial services, categorizing cyber threats into game-based (*e.g*., phishing, malware, APTs) and non-game-based (*e.g*., social engineering, compliance issues) threats. AI-driven measures for prevention and detection emphasize regular security assessments, user training, and system monitoring with incident response plans. This research provides valuable insights into the intersection of gamification and cybersecurity, offering a forward-looking perspective for both academic researchers and industry professionals.

## Introduction

In today’s world, the widespread popularity of online gaming has fueled the rise of gamification, where game design principles are applied to create meaningful positive effects at the individual level. This approach, involving the application of game design elements in non-game contexts, is widely utilized across various sectors, including banking (Raza et al., 2024), education, healthcare, transportation, and e-commerce (Avril et al., 2024). The integration of these principles has infused various aspects of life, including both academic (Kaya & Ercag, 2023) and non-academic domains (Liu et al., 2023). This phenomenon, termed “gamification,” has gathered substantial attention from academic and marketing circles, marking a paradigm shift towards incorporating interactive and engaging elements into diverse domains (Rodrigues, Costa & Oliveira, 2016). Game-based learning has proven effective in enhancing interaction at both enterprise and student levels by providing instant feedback, fostering creativity, and improving employability compared to conventional methods (Casau, Ferreira Dias & Amorim, 2023). This evolutionary trajectory also signifies a broader trend in augmenting business applications by incorporating game design principles to effectively adopt prevention techniques, spanning online gaming and more serious consideration of gaming perspectives (Bunt, Greeff & Taylor, 2024; Schlenker et al., 2018).

Gamification elements are divided into two main categories: visual and non-visual. Visual components, such as avatars and fantasy environments, are those you can see on the screen, while non-visual elements, like points, rules, and progress tracking, operate behind the scenes. Gamification plays a key role in boosting customer engagement by incorporating various features. These can be classified into achievement-based elements, like rewards, points, badges, and leaderboards (Friedrich et al., 2020; Ishaq et al., 2022) as well as immersive elements, such as avatars and fantasy settings, that enhance user experience.

*Rewards:* In the financial sector, gamified applications utilize intrinsic motivation by enabling users to achieve personal financial goals and experience a sense of mastery. At the same time, they incorporate extrinsic motivators, such as points and monetary rewards, to encourage transactions and boost user engagement (Chauhan, Akhtar & Gupta, 2021). While these rewards primarily function as external incentives, they provide limited autonomy, relying heavily on external factors. According to self-determination theory, this external regulation signifies the lowest level of self-control and personal initiative (Friedrich et al., 2020).

*Points:* In the financial sector, banks implement point accumulation in their loyalty programs to encourage online transactions and enhance users’ sense of achievement. This gamification strategy raises competition by enabling customers to complete challenges and advance through levels based on their points. Additionally, the tiered structure boosts customers’ social status within the community, further increasing engagement and loyalty (Roncone & Massari, 2022).

*Badges*: Badges are a more sophisticated feedback tool than points and leaderboards, awarded for achieving specific goals, demonstrating knowledge, or participating in knowledge-sharing activities (Friedrich et al., 2020). Banks also employ badges, which are icons that change as users progress over levels, to increase engagement. Customers are categorized into profiles based on their chosen products or services, enabling them to earn personalized badges that unlock exclusive rewards and discounts (Roncone & Massari, 2022).

*Leaderboards*: One element of games that can be integrated into a gamification strategy is the use of leaderboards. Leaderboards are designed to encourage continued use of a product or service by showing users their rankings based on points and levels. This tracking of progress not only fosters a competitive spirit but also motivates participants to improve their performance and increase repeat engagement (Roncone & Massari, 2022).

*Avatar and fantasy elements*: Visual gamification elements, such as avatars and fantasy environments, are key to creating immersive experiences. Avatars act as digital representations of users, while fantasy elements build engaging, imaginative worlds. Together, these components significantly enhance user interaction and foster a stronger personal connection within the gamified system (Tang & Zhang, 2019). In the context of banking gamification, their use can improve task performance and decision-making skills. performance and decision-making competencies by making the experience more interactive and personalize (Bitrián, Buil & Catalán, 2021; Chauhan, Akhtar & Gupta, 2021). Such strategies are widely implemented to enhance consumer engagement, promote loyalty, and shape user behavior (Putra Rahmadhan et al., 2023).

Researchers argue that maximizing the positive impact of gamification involves considering users’ characteristics. Observing various responses to the same game and the unique sense of fun for each individual, it is suggested that a universally enjoyable game is unachievable. Studies indicate significant variations in how users interpret, functionalize, and evaluate game elements, highlighting the need for personalized gamified systems (Knutas et al., 2019). Personal characteristics, such as personality and preferences, influence game elements’ enjoyment, motivation, and perceived influence. To unlock gamification’s full potential, it is important to customize systems for each person. The rapid growth of the Internet has made it a favored entertainment avenue among youth. However, rising cyber security risks within online gaming, like data breaches and attacks, are prompting developers to prioritize network and system security to improve user experience and mitigate financial risks for players (Chattopadhyay et al., 2023). The lack of understanding among users of basic online security measures, particularly in Internet banking and social media, is the primary cause of launching attacks (Fachkha, 2023). The integration of game features into business websites, known as gamification that has demonstrated efficacy in enhancing customer participation and engagement across diverse domains, including finance and education (Scholefield & Shepherd, 2019). However, the successful implementation of gamification extends beyond the mere addition of game elements, requiring a nuanced approach that actively engages users in immersive and gameful experiences to enhance awareness (Abu-Amara et al., 2024; Yasin et al., 2024; Fachkha, 2023). From a managerial standpoint, the strategic implementation of gamification in banking holds the potential to capture customer attention, drive engagement, foster retention, and contribute to the main business objectives (Jain et al., 2020; Malarout et al., 2020a). In response to the achievements of online gaming, marketers are increasingly incorporating game design principles into non-game settings, mainly focusing on captivating and retaining visitors to online platforms. A range of AI algorithms enhances gamification features, ensuring optimal decision-making strategies are implemented effectively (Onari et al., 2024). Despite these endeavors, gamification remains an experimental business strategy, underscoring a notable lack of clarity concerning customer preferences in banking. This article aims to delve into this evolving landscape, offering insights into the integration of gamification and its implications for user engagement and preferences within the e-banking sector.

### Rationale of the study

This systematic literature review (SLR) review offers a comprehensive overview of the established datasets from 2015 to 2024, addressing key research questions aimed at explaining the integration of personalized and gamification-based strategies from well-known academic sources to mitigate cyber threats in the financial sector. Detailed quality assessment criteria were also adopted to evaluate and classify the finalized studies according to their ranks. A comprehensive analysis categorizes cyber threats targeting financial institutions, discriminating between game-based and non-game-based classifications. This diverse investigation of cyber threats provides a platform for better understanding the challenges associated with financial entities along with mitigation strategies. Furthermore, this study elaborates on the best practices for detecting and preventing cyber threats within the unique gaming environments of financial institutions. Articulating these practices in an actionable manner is crucial in stimulating financial entities’ cyber security against evolving threats. Moreover, it explores the integration of artificial intelligence (AI) tools to enhance cybersecurity measures within financial sector gaming platforms. This review further explores the role of AI in mitigating cyber threats, offering visions into the cooperation between technological advancements and personalized, gamified approaches. Lastly, the multi-layered taxonomy provides a structured framework for understanding and addressing cyber threats in the gaming sector, is discussed. Table 1 illustrates the existing studies’ gamification strategies, targeted financial sector, personalization technique, and literature review category.

**Table 1:** Analysis of previous work.

Reference	Gamification strategies	Targeted financial sector	Personalization techniques	Quality assessment	SLR	Timeline	Source
Bayuk & Altobello (2019)	Yes	Yes	No	No	No	2016	Emerald
Malarout et al. (2020b)	Yes	Yes	No	No	No	2002–2019	Google Scholar
Chauhan, Akhtar & Gupta (2021)	Yes	Yes	No	Yes	Yes	2016–2020	Emerald
Tao, Akhtar & Jiayuan (2021)	Yes	Yes	Yes	No	No	2021	Google Scholar
Swacha & Gracel (2023)	Yes	No	No	No	Yes	2014–2023	MDPI
Avril et al. (2024)	Yes	No	No	No	Yes	2013–2023	Science Direct
Oliveira et al. (2023)	Yes	No	Yes	Yes	Yes	2014–2020	Springer Link
This article	Yes	Yes	Yes	Yes	Yes	2015–2024	Web of Science

The article’s structure is outlined as follows: “Methodology” provides a comprehensive review of relevant literature, explaining methodologies relating to personalized and gamification-based approaches in the context of cyber threats. “Answering the Research Question” explains the research methodology, detailing the systematic data extraction process, rigorous quality assessment criteria, and thorough inclusion/exclusion criteria. Moreover, “Taxonomy of Personalized and Gamification-based Cyber Threats” explores key questions surrounding gamification in cyber-based financial institutions, while “Limitations” introduces a taxonomy of personalized and gamification-based cyber threats faced by financial institutions. Further, “Future Direction” discusses future directions, highlighting emerging trends and areas for further investigation. Finally, “Conclusion” presents findings on the implications of gamification for financial sector cybersecurity. Overall, this study contributes to understanding the innovative ways to mitigate cyber threats and improve security in the financial sector through gamification.

### Background

This review thoroughly examines personalized gamification’s role in enhancing cybersecurity within the financial sector, offering a systematic evaluation of existing literature and explaining techniques to raise stakeholder awareness that addresses the pressing need for innovative cybersecurity measures in the financial sector. Through rigorous analysis, the review aims to uncover effective and efficient strategies to improve digital security practices. Its findings provide a valuable understanding of personalized gamification to mitigate cyber threats in finance and pave a path for future research and practical implementations.

The study of Bayuk & Altobello (2019) explored integrating gamification into financial services to enhance behavior and motivation. Through surveys targeting college students and Mechanical Turk participants, the study explores preferences for features within financial applications, revealing that former experience with such applications influences preferences for social or economic features. The study emphasizes understanding personal financial well-being and suggests that gamification could be valuable for individuals experiencing financial challenges. However, the study does not explore the personalized techniques utilized in gamification, nor does it employ an advanced research methodology, as it primarily relies on survey data. In the meantime, Malarout et al. (2020b) conducted a systematic review of gamification’s applications in various industries, focusing on its impact on customer loyalty within the banking sector. The review also identifies a positive correlation between motivation and gamification, particularly observing possible benefits in cooperative banks. Though, it does not address the personalized techniques utilized in gamification within the financial sector. Additionally, the study is not a systematic literature review but rather relies on survey data.

Chauhan, Akhtar & Gupta (2021) have thoroughly examined game-based applications in banking, highlighting its early stage of research and implementation. The study emphasized the importance of empirical research and validated methodologies, suggesting potential benefits in enhancing financial awareness levels and employee knowledge. Despite limited empirical evidence, understanding gamification’s impact on customer behavior and challenges in effective implementation are deemed crucial. Though, the study does not address the aspects of personalized gamification. According to Tao, Akhtar & Jiayuan (2021), the role of artificial intelligence (AI) has been growing in cybersecurity, stressing its importance in combating increasingly complex cyber threats driven by political and economic motives. Previous studies demonstrate AI’s effectiveness in intrusion detection systems for bolstering cybersecurity through complexity reduction and false alarm minimization. However, the study does not address the aspects of personalized gamification and does not include a quality assessment of the articles reviewed.

The study of Swacha & Gracel (2023) conducted systematic literature portraits to explore the integration of machine learning (ML) and gamification, explaining their cumulative effect on user experiences in information systems. Through extensive literature review, they uncover many domains, including the integration of gamification in machine learning education and the reciprocal relationship of machine learning in gamification research. Their analysis reveals multiple methodologies and applications, suggesting promising platforms for further exploration in amalgamating machine learning and gamification domains. However, the study does not address financial institutions, nor does it constitute a systematic literature review or include a quality assessment of the reviewed articles.

Gamification has undoubtedly generated interest in the transport and mobility sector. In their study, Avril et al. (2024) explored the broad use of gamification for its motivational and engagement benefits in this domain. The review underscores considerable variation in gamified interventions aimed at fostering safer or more eco-friendly driving, characterized by diverse methodologies and inconsistent empirical findings. Yet, the article does not address more established sectors like finance, where gamification has yielded clearer behavioral impacts, nor does it explore the use of personalization technique.

In the educational sector, the demand for gamification is also significant. Oliveira et al. (2023) emphasize the vital role of gamification in enhancing student concentration, motivation, and engagement. However, there is a pressing need for personalized gamification designs that take individual learner differences into account, as current research primarily focuses on gamer types while neglecting critical factors such as gender and demographics. This oversight highlights the need for further exploration of tailored approaches to fully realize the potential benefits of gamification in educational contexts. However, this article does not address the financial sector, it is important to note that gamification similarly plays a crucial role in that domain by enhancing user engagement and tackling cybersecurity challenges through mechanisms such as rewards and leaderboards.

Furthermore, Table 1 provides a comprehensive overview of gamification-based cyber threat studies highlighting gaps in writing style alterations and shared dataset utilization.

Numerous studies have investigated gamification strategies across various disciplines; however, there remains a significant gap in research specifically addressing the financial sector. Furthermore, many existing articles lack the incorporation of personalized techniques and fail to conduct comprehensive quality assessments. Some studies do not employ a full systematic literature review (SLR), presenting only survey-based analyses. An in-depth review should encompass all key elements, including gamification, personalized approaches, and quality evaluation, particularly within the financial sector spanning from 2015 to 2024. Each referenced article explores various gamification techniques, such as detection and prevention mechanisms, personalized approaches, and artificial intelligence tools. Notably, the table outlines the studies that incorporate an SLR approach, providing insights into the research. Additionally, it encompassing prominent academic databases such as Emerald, Google Scholar, and Multidisciplinary Digital Publishing Institute (MDPI). This comprehensive overview also helps identify gaps in gamification-based cybersecurity research within the financial sector, offering valuable insights for future investigations and expansions.

## Methodology

**Figure 1:**
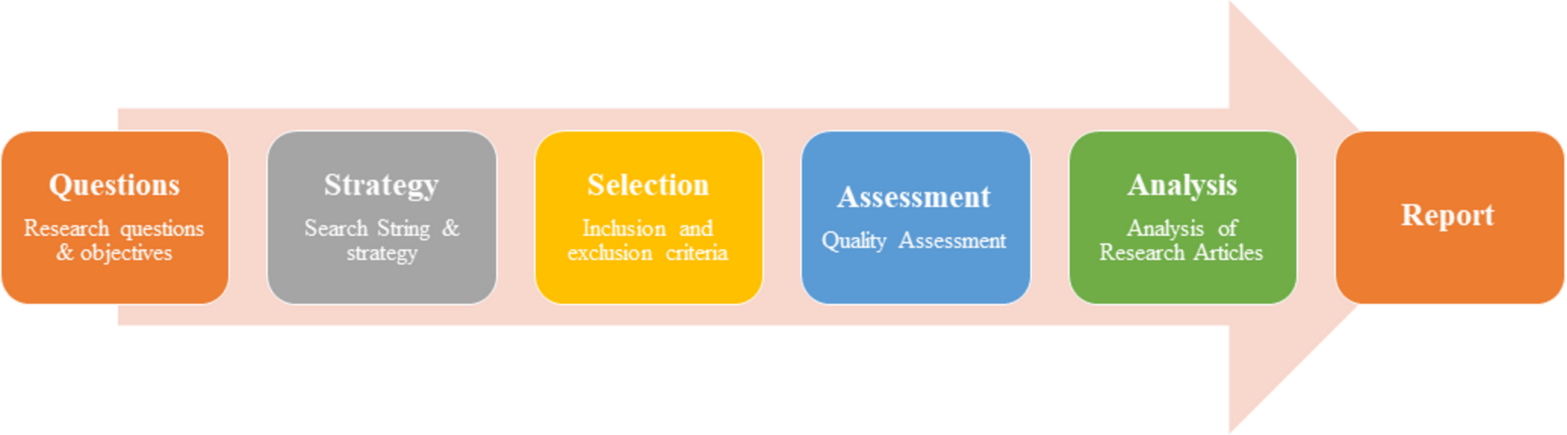
Research methodology.

This study focuses on reviewing research in personalized and gamification-based cyber threats in the financial sector domain. Insights have been extracted from various existing methodologies detailed in multiple studies (Chowdhury & Gkioulos, 2023; Bitrián, Buil & Catalán, 2021; Rodrigues et al., 2021). The aim is to comprehensively understand the paradigm of cyber threats personalized to financial organizations. Drawing upon various research methodologies, this study seeks to thoroughly analyze the challenges experienced by personalized and gamified cyber threats. Eventually, the goal is to inform effective strategies for mitigating these threats within the financial sector (Bayuk & Altobello, 2019; Chauhan, Akhtar & Gupta, 2021; Malarout et al., 2020b; Swacha & Gracel, 2023; Tao, Akhtar & Jiayuan, 2021). The researcher has formulated clear research objectives and devised appropriate research questions along with the search strategy. This approach allows us to effectively search for and identify relevant articles on gamification-based cyber threats for financial institutions, for which the flow is given in Fig. 1.

The formulated questions will deal with the gamification-based cyber threats in financial institutions efficiently, and each is designed to explore specific aspects of personalized gamification. The current review has shed light on the various cyber threats and is focused on the taxonomy of this domain as well. The research questions presented in Table 2 are as follows:

**Table 2:** Research questions.

RQ	Statements	Objective
RQ1	What reputable academic sources were utilized to collect scholarly articles, and what methodology was employed to assess the quality of the selected articles on applying gamification to mitigate cyber threats in financial institutions?	It will ensure reliable, high-quality research findings for informed decision-making, cybersecurity, and financial technology advancement.
RQ2	What is the classification of game-based and non-game-based cyber threats aimed at financial institutions?	It will enhance understanding and develop tailored mitigation strategies for safeguarding financial systems.
RQ3	What are the best practices for detecting and preventing cyber threats within financial institutions’ game-based environments, and how can these practices be articulated skillfully?	The best practices for detecting and preventing cyber threats within game-based environments aim to provide clear guidance on effective strategies.
RQ4	How and which AI tools be strategically integrated to enhance cybersecurity measures and mitigate cyber threats within gaming platforms in the financial sector?	The strategic integration of AI tools to detect and mitigate cyber threats effectively ensures financial systems and data security and integrity.

### Research strategy

The review is constructed to explore personalized and gamification-based cyber threat parameters within financial institutions. This study innovatively analyses cyber threats within gamified environments with a rigorous academic approach. By focusing on integrating game design principles into cybersecurity strategies, this research aims to offer novel insights into mitigating threats in financial setups. The innovative aspect lies in its academic rigor in understanding cyber threats within gamified contexts. This work advances scholarly understanding of cybersecurity in the digital age, particularly in financial domains where gamification strategies are increasingly employed (Manzoor et al., 2023). The following search string is used to find relevant articles for this study.

(“personalized gamification” OR “gamified attacks” OR “gamification”) AND (“cyber threats” OR “cyber security” OR “cyber-attacks”) AND (“financial institutions” OR “banks” OR “financial services” OR “financial corporations” OR “investment firms” OR “insurance companies” AND (“security breaches” OR “vulnerabilities” OR “attacks”) AND (“techniques” OR “methods” OR “approaches” OR “algorithms”) AND (“evolution” OR “development” OR “advancements” OR “progress”) AND (“challenges” OR “limitations” OR “issues” OR “obstacles”)

Searching for primary studies in gamification-based cyber threats involves collecting articles from diverse sources, including the Institute of Electrical and Electronics Engineers (IEEE), Springer, Elsevier, Emerald, Association for Computing Machinery (ACM), and other reputable channels. Search strings were drawn onto the downloaded articles from reliable and recognized search engines and databases. Full texts and downloaded abstracts were explored, and 48 articles were selected.

### Study selection

**Figure 2:**
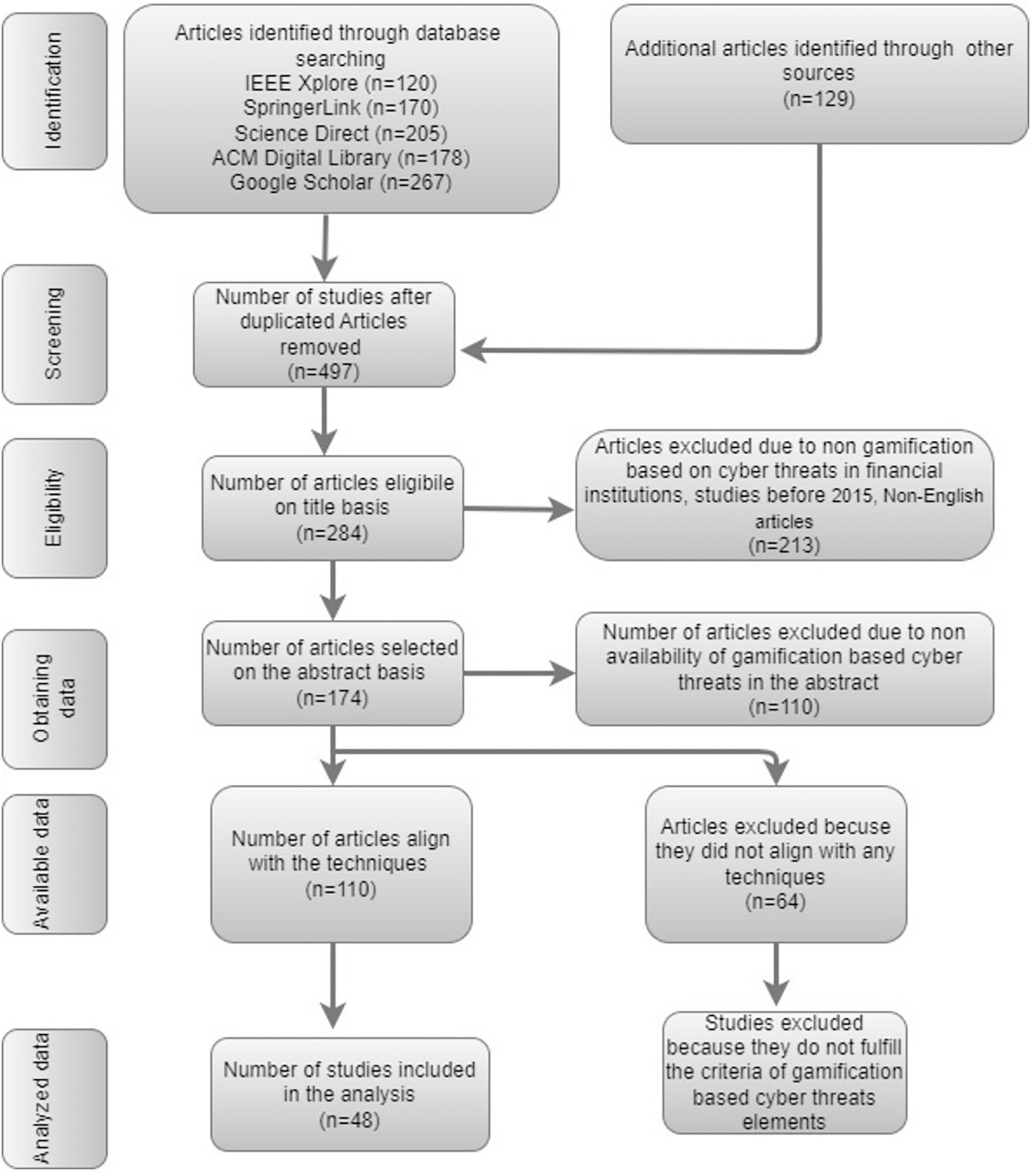
Study selection criteria.

The study selection is a critical step in the systematic literature review process. It involves reviewing the titles and abstracts of the articles obtained through the search strategy to identify relevant studies that meet the inclusion and exclusion criteria. This step aims to reduce the number of articles to a manageable level while retaining those most likely to provide useful information. Figure 2 portrays the steps involved in the study selection of the articles and how articles are included and excluded from the selected criteria. This portraited that 497 articles were initially identified from multiple databases, including IEEE Xplore, SpringerLink, ScienceDirect, ACM Digital Library, and Google Scholar, along with additional sources. After removing duplicates, 284 articles were eligible based on their titles. Studies were then screened for relevance, focusing on gamification-based approaches to address cyber threats in financial institutions, with only English-language articles published after 2015 being considered. Following an abstract review, 174 articles remained, but 110 were excluded for not focusing on gamification and cyber threats. Ultimately, 64 more articles were excluded due to misalignment with specific techniques of interest, leaving 48 studies for final analysis.

For this purpose, the researchers did a systematic literature review from 2015 to 2024 to perform gamification-based cyber threats for financial institutions. The selection process is performed by defined Inclusion and exclusion criteria in Table 3 as follows:

**Table 3:** Inclusion and exclusion criteria.

Criteria	Inclusion criteria	Exclusion criteria
IE1	Studies focusing on gamification based cyber threats in financial institutions	Studies not related to gamification based cyber threats in financial institutions.
IE2	Articles were published in a journal or well-known conference between 2015 to 2024	Articles were not published in a journal or well-known conference between 2015 to 2024
IE3	Research articles written only in English	Non-English articles
IE4	Articles published in peer-reviewed journals, conference proceedings, or reputable sources.	Non-peer-reviewed sources or articles from unreliable or questionable publishers.
IE5	Studies that provide detailed information on the techniques employed on gamification based cyber threats in financial institutions	Studies lack sufficient information on the methodologies or techniques employed on gamification based cyber threats in financial institutions.

In this study, 1,069 initial studies were retrieved from multiple sources for gamification-based cyber threats in financial institutions. The selection process involved shortlisting the articles based on predefined inclusion and exclusion criteria.

- *Search string:* At the initial stage, articles collected from various databases are carefully eliminated. Many irrelevant articles remained at this point. After this step, only 1,069 articles remained.
- *Duplicated articles:* At this stage, duplicate articles must be eliminated and acquired from diverse sources, leaving only 497 articles available for further analysis.
- *Title-based search:* In the third stage, unrelated articles based on their title are carefully eliminated. At this point, there were many irrelevant articles. After this step, only 284 articles remained.
- *Abstract-based search:* This step excludes articles based on the abstracts of the articles selected in the initial stage. The articles are organized for analysis and research methodology. After this point, just 174 articles were left for further analysis.
- *Technique text-based analysis:* At this stage, the quality of the articles is assessed. The study’s analysis was done using any technique implemented in the article. A total of 110 articles were selected for further analysis.
- *Full text-based analysis:* In the final stage, the empirical quality of the articles chosen earlier is assessed. A comprehensive text analysis of the study has been done. A total of 48 articles were selected from 110 articles.

The study of gamification and cybersecurity in financial institutions, drawing from 48 precisely selected articles across valued databases and journals. Each source was analyzed for methodological rigor, relevance, authoritativeness, and publication credibility, ensuring the highest standards of academic integrity.

### Study selection results—based on publishers

A thorough review was undertaken on a *corpus* of 48 articles to clarify the research questions posed earlier. Table 4 provides an insightful breakdown of the distribution of studies according to their sources across diverse platforms, including renowned publishers such as IEEE, Springer, Elsevier, Emerald, ACM, and other notable platforms.

**Table 4:** Study selection results—based on publishers.

Data extract	Search string	Duplicated	Title	Abstract	Technique	Full text
IEEE	120	97	38	25	14	5
Springer	170	84	60	34	20	8
Elsevier	205	62	48	37	27	14
ACM	178	95	54	17	9	2
Emerald	267	76	36	29	15	6
Others	129	83	48	32	25	13
Total	1,069	497	284	174	110	48

**Figure 3:**
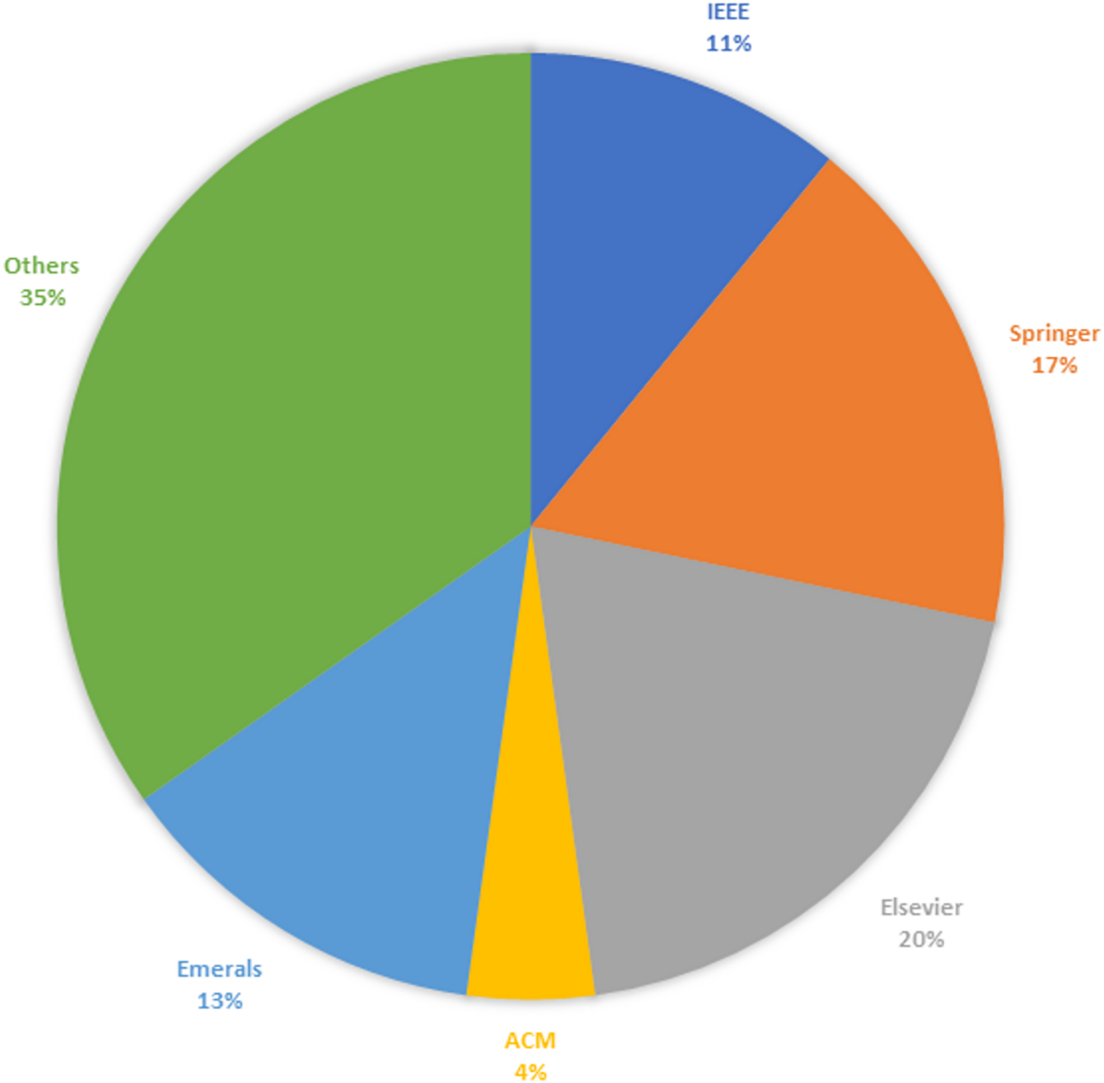
Study selection results.

Figure 3 shows the percentage of full-text articles gathered from well-known platforms, such as IEEE, Springer, Elsevier, ACM, and others. Some relevant research articles that were vital in this SLR were also included.

## Answering the research question

Gamification is a strategy incorporating game-based elements into a non-game perspective that has emerged in user engagement and motivation in this versatile field. In the domain of cyber-based financial institutions, where digital interfaces dominate interactions, the integration of gamification principles holds significant importance. This section investigates key elements surrounding the application, impact, and challenges associated with the gamification platform within cyber-financial environments. Through a systematic exploration of these questions, the implications of gamification are to redesign the user experiences and behaviors within financial technology.

**RQ1: What reputable academic sources were utilized to collect scholarly articles, and what methodology was employed to assess the quality of the selected articles on applying gamification to mitigate cyber threats in financial institutions?**

Gamification effectively strengthens cybersecurity measures within financial institutions, specifically regarding the quality and reliability of academic sources informing research activities. This section outlines the thorough process employed to enumerate reputable scholarly articles and the methodology for assessing their quality. By scrutinizing and selecting relevant academic sources and employing robust quality assessment criteria, this study ensures a comprehensive understanding of the application of gamification in mitigating cyber threats within the financial sector.

### Study selection results—based on journals/others

A comprehensive analysis was conducted on 48 articles to address the research questions outlined earlier. Table 5 shows the distribution of studies according to their sources, encompassing various platforms such as journals, reputable books, arXiv preprints, and reports. This categorization allows for a thorough examination of the diverse sources contributing to understanding the subject matter.

**Table 5:** Journal or conferences or others.

Reference	Journal/Others	Frequency
Rodrigues, Costa & Oliveira (2016), Baptista & Oliveira (2019)	Computers in Human Behavior	2
Bayuk & Altobello (2019), Bitrián, Buil & Catalán (2021)	International Journal of Bank Marketing	2
Swacha & Gracel (2023), Mishra (2023)	Applied Sciences	2
Yasin et al. (2019), Angafor, Yevseyeva & He (2020)	Security and Privacy	2
Abu-Amara et al. (2021), Abu-Amara et al. (2024)	International Journal of Information Technology	2
Yasin et al. (2024)	Information and Software Technology	1
Onari et al. (2024)	Knowledge-Based Systems	1
Chauhan, Akhtar & Gupta (2021)	Young Consumers	1
Yasin et al. (2019)	IET Software	1
Knutas et al. (2019)	Multimedia Tools and Applications	1
Hart et al. (2020)	Computers and Security	1
Rodrigues et al. (2021)	Proceedings of the ACM on Human-Computer Interaction	1
Van der Heide & Želinský (2021)	Journal of Cultural Economy	1
Luh et al. (2020)	Journal of Computer Virology and Hacking Techniques	1
Kävrestad et al. (2022)	Future Internet	1
Malarout et al. (2020b)	Test and Engineering Management	1
Ashta & Herrmann (2021)	Strategic Change	1
Rahi & Abd. Ghani (2019)	The International Journal of Information and Learning Technology	1
Singh (2021)	Turkish Journal of Computer and Mathematics Education	1
Chowdhury & Gkioulos (2023)	International Journal of Information Security	1
Zeadally et al. (2020)	IEEE Access	1
Mittal et al. (2021)	International Journal of Information Management Data Insights	1
Rawindaran, Jayal & Prakash (2021)	Computers	1
Tao, Akhtar & Jiayuan (2021)	EAI Endorsed Transactions on Creative Technologies	1
Erbaşı, Tural & Çoşkuner (2023)	Orclever Proceedings of Research and Development	1
Wanick & Bui (2019)	International Journal of Serious Games	1
Lai & Langley (2024)	Geoforum	1
Silic & Lowry (2020)	Journal of Management Information Systems	1
Chattopadhyay et al. (2023)	International Conference on Advance Computing and Innovative Technologies in Engineering (ICACITE)	1
Scholefield & Shepherd (2019)	HCI for Cybersecurity, Privacy, and Trust: First International Conference	1
Hale, Gamble & Gamble (2015)	Hawaii International Conference on System Sciences	1
Das & Sandhane (2021)	Journal of Physics: Conference Series	1
Maleh et al. (2021)	Artificial Intelligence and Blockchain for Future Cybersecurity Applications	1
Schlenker et al. (2018)	International Conference on Autonomous Agents and Multiagent Systems	1
Datta, Whitmore & Nwankpa (2021)	The big data-driven digital economy: Artificial and computational intelligence	1
Roy & Jain (2022)	Management and Information Technology in the Digital Era: Challenges and Perspectives	1
Kurmanova, Kurmanova & Nurdavliatova (2020)	International Scientific Conference “Far East Con”	1
Cook et al. (2016)	International Symposium for ICS and SCADA Cyber Security Research	1
Hossain et al. (2022)	International Conference on Informatics, Multimedia, Cyber and Information System (ICIMCIS)	1
Gjertsen et al. (2017)	ICISSP	1
Hanif (2021)	arXiv preprint arXiv:2112.08441	1
Drewniak & Posadzińska (2020)	University of Piraeus. International Strategic Management Association	1
Zysman, Nitzberg & On (2020)	BRIE Working Paper	1
**Total = 48**		

### Quality assessment

**Figure 4:**
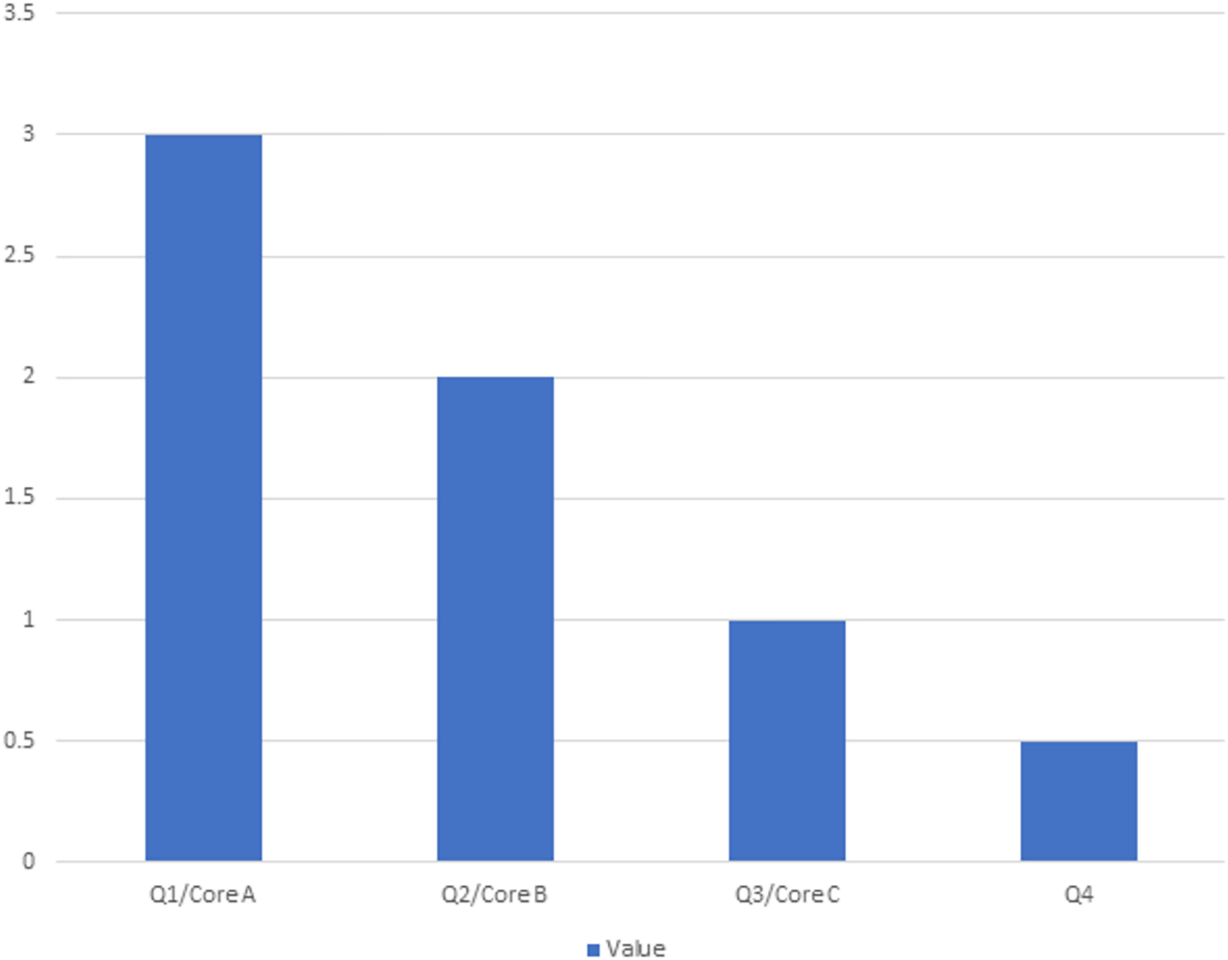
Score pattern of publication channels.

The quality of the SLR is analyzed by rating each study on a score of 3–0.5. The research study has been scored against each of the four dimensions, and a score of 3 represents high, a rating of 2 represents medium, 1 represents average, and a score of 0.5 represents the low rank of the article, as illustrated in Fig. 4.

#### Quality assessment criteria

The following are the criteria used to assess the quality of the selected primary studies. This quality assessment was done on the following basis:

- The study focuses on the Integration and Analysis of the data, and the possible answers were: Yes (1), No (0)
- Assessment: 2021, 2022, 2023, 2024 = (3), 2018, 2019–2020 (2), 2016, 2017 (1), before 2015 (0.5)
- The study is published in a prestigious venue and assessed based on the CORE ranking for conferences (A, B, and C) and the Scientific Journal Ranking (SJR) for journals, letters, and scientific reports, categorized into Q1, Q2, Q3, and Q4. articles in the Q1/Core A category receive a score of 3, Q2/Core B category articles score 2, Q3/Core C category articles are rated at 1, and Q4 articles receive a score of 0.5.
- The articles are cited more than 200 (3), 200–150 (2.5), 149–100 (2), 99–50 (1.5), 49–1 (1).

The evaluation criteria outlined in the aforementioned bullet points incorporate the analysis of data (W), yearly disruption of articles (X), articles’ ranking (Y), and citation frequency (Z), as detailed in Table 6. This table offers a comprehensive overview of the quality assessment conducted for each article, specifying their titles and sources, including journals (J), conferences (C), books (B), ArXiv (A), or reports (R). The major aim is to assess the credibility of the included articles, which reflects various quality indicators like methodological consistency, theoretical framework, and alignment with research objectives. Through meticulous scrutiny, Table 6 aims to provide readers with insights into the reliability and validity of the findings presented in each article.

**Table 6:** Quality assessment.

Ref.	Research type	Methodological tool	Year	Journal/Conference/Book/ArXiv/Report	Quality assessment				
					W	X	Y	Z	Total
Yasin et al. (2024)	Mixed	Experimental and survey findings	2024	J	1	3	3	1	8
Abu-Amara et al. (2024)	Mixed	Experimental and survey findings	2024	J	1	3	2	0	6
Onari et al. (2024)	Mixed	Experimental and Discussion	2024	J	1	3	3	0	7
Chattopadhyay et al. (2023)	Mixed	Statistical analysis and survey	2023	C	1	3	0	1	5
Swacha & Gracel (2023)	Qualitative	Systematic literature review	2023	J	1	3	2	1	7
Mishra (2023)	Quantitative	Experimental approach	2023	J	1	3	2	1	7
Chowdhury & Gkioulos (2023)	Mixed	Experimental and survey findings	2023	J	1	3	3	1	8
Erbaşı, Tural & Çoşkuner (2023)	Qualitative	Survey	2023	J	1	3	0	0	4
Lai & Langley (2024)	Qualitative	Case studies and discussions	2023	J	1	3	3	1	8
Kävrestad et al. (2022)	Quantitative	Experimental and Statistical Analysis	2022	J	1	3	2	1	7
Roy & Jain (2022)	Qualitative	Market Trends	2022	B	1	3	0	1	5
Hossain et al. (2022)	Mixed	Comprehensive strategies and statistics analysis	2022	B	1	3	0	1	5
Bitrián, Buil & Catalán (2021)	Mixed	Literature review, questionnaire, and statistical analysis	2021	J	1	3	2	1	7
Rodrigues et al. (2021)	Mixed	Interviews and data analysis	2021	J	1	3	3	1	8
Chauhan, Akhtar & Gupta (2021)	Mixed	Literature review and statistical analysis	2021	J	1	3	3	1	8
Van der Heide & Želinský (2021)	Qualitative	Research Approach	2021	J	1	3	3	1	8
Das & Sandhane (2021)	Qualitative	Literature review and discussion	2021	B	1	3	0	1	5
Maleh et al. (2021)	Qualitative	Literature review and discussion	2021	B	1	3	0	1	5
Ashta & Herrmann (2021)	Qualitative	Literature review and discussion of case studies	2021	J	1	3	2	2	8
Singh (2021)	Qualitative	Literature review and discussion	2021	J	1	3	0	1	5
Datta, Whitmore & Nwankpa (2021)	Mixed	Literature review and experimental approach	2021	B	1	3	0	1	5
Mittal et al. (2021)	Qualitative	Propose the design of game	2021	J	1	3	3	1	8
Rawindaran, Jayal & Prakash (2021)	Mixed	Discussion and statistical analysis	2021	J	1	3	2	1	7
Tao, Akhtar & Jiayuan (2021)	Qualitative	Discussion	2021	J	1	3	0	1	5
Hanif (2021)	Mixed	Experimental and evaluation	2021	A	1	3	0	1	5
Abu-Amara et al. (2021)	Mixed	Discussion and survey results statistics	2021	J	1	3	2	1	7
Hart et al. (2020)	Mixed	Discussion and statistical analysis	2020	J	1	2	3	2.5	8.5
Luh et al. (2020)	Mixed research	Discussion and statistical analysis	2020	J	1	2	3	1	7
Malarout et al. (2020b)	Qualitative	Suggestion and statistics	2020	J	1	2	0	1	4
Angafor, Yevseyeva & He (2020)	Qualitative	Discussion	2020	J	1	2	0	1	4
Zysman, Nitzberg & On (2020)	Qualitative	Discussion and statistical analysis	2020	R	1	2	0	1	4
Zeadally et al. (2020)	Qualitative	Discussion	2020	J	1	2	3	2.5	8.5
Kurmanova, Kurmanova & Nurdavliatova (2020)	Mixed	Discussion and statistical analysis	2020	B	1	2	0	1	4
Silic & Lowry (2020)	Mixed	Discussion and statistical analysis	2020	J	1	2	3	2.5	8.5
Drewniak & Posadzińska (2020)	Mixed	Hypothesis testing and survey	2020	R	1	2	0	1	4
Schlenker et al. (2018)	Quantitative	Experimental approach	2018	C	1	2	3	2	8
Scholefield & Shepherd (2019)	Mixed	Discussion and statistical analysis	2019	B	1	2	3	1.5	7.5
Yasin et al. (2019)	Mixed	Survey and statistical analysis	2019	J	1	2	2	1.5	6.5
Knutas et al. (2019)	Mixed	Discussion and implementation of algorithms	2019	J	1	2	3	1.5	7.5
Bayuk & Altobello (2019)	Mixed	Survey and statistical analysis	2019	J	1	2	2	2	7
Yasin et al. (2019)	Qualitative	Literature review and interviews	2019	J	1	2	0	1	4
Baptista & Oliveira (2019)	Quantitative	Statistical analysis	2019	J	1	2	3	3	9
Rahi & Abd. Ghani (2019)	Quantitative	Statistical analysis	2019	J	1	2	2	2	7
Wanick & Bui (2019)	Mixed	Systematic review and Statistical results	2019	J	1	2	1	1.5	5.5
Gjertsen et al. (2017)	Qualitative	Discussion	2017	B	1	3	0	1	5
Rodrigues, Costa & Oliveira (2016)	Qualitative	Survey and Questionnaire	2016	J	1	1	3	2	7
Cook et al. (2016)	Qualitative	Discussion	2016	B	1	1	0	1	3
Hale, Gamble & Gamble (2015)	Mixed	Discussion and statistical analysis	2015	C	1	0.5	0	1	2.5

Table 7 presents an overview of the quality assessment of the articles’ scores for focusing on game-based cyber threats in financial institutions. The scores range from 9, which is the highest, to 2.5, the lowest. This evaluation is also necessary to ensure the rigor and reliability of the studies addressing game-based cyber threats in financial institutions. Each score is assigned by a list of references corresponding to the articles evaluated. The scores are derived from rigorous criteria, where the score is multiplied by the total number of articles evaluated under the scoring criteria.

**Table 7:** Overall quality assessment scores.

References	Score	Scoring criteria	Total
Baptista & Oliveira (2019)	9	9 * 1	9
Gjertsen et al. (2017), Hart et al. (2020), Lai & Langley (2024)	8.5	8.5 * 3	25.5
Abu-Amara et al. (2021), Chauhan, Akhtar & Gupta (2021), Chowdhury & Gkioulos (2023), Datta, Whitmore & Nwankpa (2021), Rawindaran, Jayal & Prakash (2021), Rodrigues et al. (2021), Schlenker et al. (2018), Van der Heide & Želinský (2021), Yasin et al. (2024)	8	8 * 9	72
Knutas et al. (2019), Scholefield & Shepherd (2019)	7.5	7.5 * 2	15
Ashta & Herrmann (2021), Bayuk & Altobello (2019), Hossain et al. (2022), Kävrestad et al. (2022), Bitrián, Buil & Catalán (2021), Mishra (2023), Onari et al. (2024), Rodrigues, Costa & Oliveira (2016), Silic & Lowry (2020), Swacha & Gracel (2023), Zeadally et al. (2020)	7	7 * 11	77
Yasin et al. (2019)	6.5	6.5 * 1	6.5
Yasin et al. (2024)	6	6 * 1	6
Maleh et al. (2020)	5.5	5.5 * 1	5.5
Bayuk & Altobello (2019), Das & Sandhane (2021), Drewniak & Posadzińska (2020), Erbaşı, Tural & Çoşkuner (2023), Hanif (2021), Singh (2021), Kurmanova, Kurmanova & Nurdavliatova (2020), Mittal et al. (2021), Rahi & Abd. Ghani (2019), Tao, Akhtar & Jiayuan (2021)	5	5 * 10	50
Angafor, Yevseyeva & He (2020), Hale, Gamble & Gamble (2015), Malarout et al. (2020b), Roy & Jain (2022), Wanick & Bui (2019), Yasin et al. (2019), Zysman, Nitzberg & On (2020)	4	4 * 7	28
Cook et al. (2016)	3	3 * 1	3
Luh et al. (2020)	2.5	2.5 * 1	2.5

Scoring criteria score * Number of articles.

This systematic assessment provides relative quality and distribution of research contributions in this domain.

**RQ2: What is the classification of game-based and non-game-based cyber threats aimed at financial institutions?**

The research question aims to classify cyber threats into game-based and non-game-based categories, specifically focusing on those targeting financial institutions. It pursues to understand various types of threats and their respective impacts on financial systems and institutions. This classification helps to identify specific vulnerabilities and develop targeted strategies for mitigating cyber risks in financial institutions.

### Game-based cyber threats

In the digitalization world, the advantages and disadvantages include digital crimes and real-world threats. Within this perspective, gamification emerges as a crucial element in bringing awareness campaigns to educate the public about cyber threats. Game-based approaches to understanding cyber threats, specifically in financial domains. These initiatives employ a strategic, comprehensive methodology to suggest a structured game plan, often incorporating sophisticated strategies like advanced persistent threats (APTs) (Luh et al., 2020), social engineering strategies (Yasin et al., 2019), and targeted assaults designed to penetrate sensitive financial systems or their associated networks.

#### Advanced persistent threats

In the domain of gamification, where persistent cyber-attacks continue to increase effective defence mechanisms to ensure the continuous operation of networks are becoming increasingly pressing. Luh et al. (2020) introduced PenQuest, an innovative gamified attacker/defender metamodel designed for the workings of cyber-attacks. The framework also brings awareness initiatives, thorough risk evaluation protocols, and the utilization of serious games like PenQuest to replicate authentic attack scenarios. Also, PenQuest integrates industry standards to incorporate APT campaigns, providing a valuable tool for risk assessment actions and giving educational resources in multiple situations. The study also highlights PenQuest’s potential for rigorous connections between security protocols and realistic defence strategies, thus improving the overall cybersecurity platform (Luh et al., 2022).

#### Social engineering

Similarly, cyber-attack responsibility often requires human involvement through unintentional misconfigurations or a lack of awareness among users (Sharma & Thapa, 2023; Fatokun Faith, Long & Hamid, 2024; Fachkha, 2023; Yasin et al., 2019) explained tactics like eavesdropping and shoulder surfing, shedding light on the challenges individuals face in discriminating against these threats, which impact both game-based and non-game-based cyber vulnerabilities in financial institutions. Additionally, the study advocates for game-based analyses to enhance understanding of these attacks from a gaming perspective, underscoring the urgent need to enhance cybersecurity education within the financial sector’s gaming platform (Luh et al., 2020). The gaming environment activities raise awareness regarding cyber threats to lessen human negligence, offering educational resources and guidance in this domain.

### Non-game-based cyber threats

On the other hand, non-game-based cyber threats incorporate a broader range of attacks, including malware infections, ransomware campaigns, distributed denial-of-service (DDoS) attacks, and insider threats. These threats exploit vulnerabilities in security infrastructure and human factors and pose significant risks to financial institutions.

#### Phishing attacks

Non-game-based threats, illustrated in studies like Hale, Gamble & Gamble (2015), Kävrestad et al. (2022) involve various phishing techniques to deceive individuals into disclosing personal information or installing malware. For instance, Chattopadhyay et al. (2023) highlighted widespread cyber security risks intrinsic to gaming platforms, like data and identity theft, phishing exploits, and malware intrusions, highlighting the financial consequences for affected players. These sorts of attacks exploit human psychology and due to a lack of digital awareness, often lead to data breaches or unauthorized access that looks like it is from a legitimate channel (Yasin et al., 2024).

#### Malware infections

In addition to phishing attacks, malicious actors posed a significant threat in non-game-based contexts by exploiting vulnerabilities to compromise systems and sensitive data (Yasin et al., 2024). This attack incorporates harmful software elements designed to deceive users and facilitate unauthorized access to confidential information, as demonstrated by relevant research studies explaining non-game-based cybersecurity threats. Further, the incorporation of gamification techniques into non-gaming sectors, like financial institutions engaging in credit card rewards systems, is intended to enhance user engagement. But this integration also brings forth security concerns restricting potential vulnerabilities that cybercriminals may exploit to achieve their motives (Scholefield & Shepherd, 2019). Additionally, gamification software development in e-banking systems highlights the role of users in testing and improving software, with findings indicating design, appearance, functionality, rules, and objectives in the development of the software (Chattopadhyay et al., 2023).

Therefore, the intersection of digitalization and gamification within the financial sector presents various challenges in efficiently impeding the way of cyber threats (Sharma & Thapa, 2023). Game-based threats highlight strategic complexities, while non-game-based attacks exploit vulnerabilities in security infrastructure and human factors. Incorporating gamification in financial institutions aims to improve customer engagement and bring out security vulnerabilities. Table 8 headings offer a comprehensive overview of cyber-attacks within the gamification environment because it covers the source of research, the nature of attacks, parameters outlining both game and non-game contexts, research goals, notable findings, and study limitations.

**Table 8:** Classification of cyber-threats.

Reference	Aim/Attack	Non-game-based parameters	Context game-based parameters	Purpose of research	Key findings	Limitation
Scholefield & Shepherd (2019)	Gamification and bank customers to use credit cards (credit card attack)	It enhances satisfaction but cannot contribute to the influence level of customers	Game-based environment (reward system)	Impact of gamification, in reward points, on bank customers’ engagement, satisfaction with credit card usage	Gamification improves engagement and satisfaction; it does not significantly influence customer intention.	Many bank colleagues did not know what gamification was, and the sample size was small.
Hale, Gamble & Gamble (2015)	Develop effective and realistic strategies for phishing attacks (Phishing attack)	Educational content, training module, testing components, user progress tracking	Simulation platform (game-like environment), game-based scenario of phishing attack, gamification techniques (Points, levels, rewards)	Address the challenges linked with existing approaches to combat phishing attacks and evaluate a novel method that combines game-based learning techniques	Outlines a structured three-phase experiment on the Cyber Phishing simulation platform and systematically evaluates the proposed game-based learning approach	Issues with test-based techniques (adjustment of behavior by subjects) and in-the-wild studies (lack of consent or data exposure)
Yasin et al. (2019), Sharma & Thapa (2023), Yasin et al. (2019)	Contribute to understanding social engineering (SE) attacks	Interview session, educational content on real-world examples	Game-based environment (goals, players, characters, rewards, rules), and it brings an educational approach	Address the increasing occurrence of social engineering attacks and the challenges people face in identifying and understanding these attacks	Various types of social engineering attacks, simulation of game-based analysis, and raising awareness level	Long-term assessment to determine the continual impact of the game-based method on participants makes it challenging to generalize the findings to broader populations
Luh et al. (2020, 2022)	Activities of reconnaissance, exploitation, social engineering, state manipulation, API abuse, brute force, illegal access, and data manipulation	Incorporation of established security standards, taxonomies, and the utilization of external data sources	Game-based environment (various actors, assets, and actions to allow the evaluation of cyber risks)	Contribute to the advancement of cybersecurity training methodologies by introducing a flexible and comprehensive gamified model, PenQuest	Gamified model PenQuest forms the basis for understanding targeted attacks by combining threat intelligence and monitoring data in information security.	This model’s reliance on external data sources leads to potentially outdated patterns that challenge attack mapping and control assignment.
Kävrestad et al. (2022)	Phishing emails	Aiming to increase awareness and improve user behavior	Interactive and engaging nature to play a game, potentially enhancing user learning	Assesses the effectiveness of to enhance users’ ability to identify phishing emails	Results indicate that both training methods improve user behavior, with CBMT showing a higher degree of effectiveness	Participant numbers are less
Chattopadhyay et al. (2023)	Corporate Account Takeover (CATO), ATM Cash Out, and plug-in software	Bring awareness and recommendations	Popular PC games are exploited as bait for cyber-attacks, manipulating web-based control panels to disrupt gameplay and exploit susceptibility to malware threats.	Game developers have become aware of cyber security concerns in online games, such as information leaks and cyberattacks.	Cybercriminals manipulate web-based control panels of ATMs, impacting medium and small banking institutions.	Insufficient coverage of mobile gaming threats and no insight into emerging cyber threats in the industry
Rodrigues et al. (2021)	Explore users’ design preferences in gamified e-banking software to improve customer experience.	Aiming to increase awareness and improve user behavior	Incorporating elements like points, badges, leaderboards, and rewards, e-banking platforms	Designing gamified software in the e-banking sector, with a focus on understanding users’ design preferences	Emphasis on regulatory standards, usability, and transparency in building trust and loyalty among e-banking user	Influenced by cultural factors

The studies collectively examine the impact of gamification in the cybersecurity and banking sector, aiming to enhance user engagement, behavior, and awareness level, though each faces specific limitations. Scholefield & Shepherd (2019) found that gamification increased customer satisfaction but did not meaningfully influence customer intent, with limitations due to a small sample size and participants’ lack of knowledge about the gamification environment. Hale, Gamble & Gamble (2015) developed game-based strategies for phishing prevention but faced various challenges with test-based techniques and ethical issues in the wild studies, where participant consent and data exposure were concerns. Yasin et al. (2019) used game-based simulations to raise awareness of social engineering attacks, but the results were hard to generalize due to the absence of long-term assessments. Moreover, Luh et al. (2020) introduced the PenQuest model for cybersecurity training, combining threat intelligence with gamified learning, though its reliance on external data sources risked using outdated attack patterns. Kävrestad et al. (2022) explored game-based phishing detection training, showing improvements in user behavior, but the small sample size limited the study’s broader applicability. Similarly, Chattopadhyay et al. (2023) highlighted cyber-attacks in gaming and banking, though it lacked coverage of mobile gaming threats and emerging cyber risks. Rodrigues et al. (2021) explored users’ design preferences for gamified e-banking software, emphasizing the importance of usability, transparency, and regulatory standards in building trust and loyalty, but noted that the findings were influenced by cultural factors, which may limit generalization across different populations.

**RQ3: What are the best practices for detecting and preventing cyber threats within financial institutions’ game-based environments, and how can these practices be articulated skillfully?**

The advent of gamification within financial institutions presents novel challenges for ideal cybersecurity protocols. It demands a multilayered approach that merges technological innovations with comprehensive employee training. Gamification platforms can reinforce institutions’ defensive strategies by addressing cybersecurity threats.

### Preventing measures for game-based cyber threats

Cybersecurity deals with a comprehensive approach to counter cyber threats in the rapid expansion of the integration of gaming and finance. This includes implementing rigorous training and awareness with the knowledge and skills necessary for identifying and mitigating risks. Furthermore, establishing crisis management programs and incident response frameworks is essential to ensure prompt and efficient resolution of security breaches. Such multi-layered approaches are necessary for safeguarding financial institutions against the emerging landscape of cyber threats.

#### Training and awareness of technological solutions

The cybersecurity platform is crucial to adopt holistic strategies that effectively struggle against evolving threats. In gamified simulations, financial institutions can conduct regular security assessments, providing huge experience to evaluate and enhance their defences by utilizing multi-layered defence strategies by combining technological solutions like endpoint and network security measures, penetration testing, and threat intelligence feeds (Luh et al., 2020). Additionally, integrating game-based training modules like Context-Based Micro-Training (CBMT) authorizes users with interactive learning experiences, development of awareness levels, and proactive responses against phishing attacks (Kävrestad et al., 2022). Furthermore, Chowdhury & Gkioulos (2023) discussed the development of personalized learning theory-based models to customize cybersecurity training exercises to individual requirements, maximizing effectiveness and ensuring a strong defence that hinders the way of cyber threats.

#### Crisis management and incident response

In the environment of an organization, an effective strategy includes taking training and awareness initiatives to train individuals with the required skills to utilize technological solutions appropriately. This involves differentiating potential cybersecurity threats and expertly responding to them in adverse incidents. To reinforce preparedness, it is essential to construct comprehensive incident response plans completed by scenario-based training methodologies (Angafor, Yevseyeva & He, 2020). Additionally, Chattopadhyay et al. (2023) advocated for fostering collaboration between game developers and cybersecurity experts to stay updated on evolving risks that ensure a proactive approach to cybersecurity in gaming platforms. These plans ensure efficient and effective responses to security breaches, minimizing the impact on financial institutions and enabling timely responses to protect sensitive data and information.

#### Regulatory compliance and vendor management

Crisis management and incident response domains are fundamentally interconnected with regulatory compliance, forming a holistic platform that expertly navigates cyber threats while addressing regulatory requirements. Kävrestad et al. (2022) emphasized using gamified training modules to ensure regulatory compliance and employee compliance with standards in financial institutions. Integrating gamification enhances engagement and retention of compliance rules among employees. This proactive approach reinforces cybersecurity, safeguarding financial data and operations against potential threats. These prevention measures highlight the importance of a multi-layered approach merging technological solutions, employee training, crisis management, and regulatory compliance to mitigate cyber threats in financial institutions successfully.

### Detection measures for game-based cyber threats

Detection measures are crucial to address game-based cyber threats within financial institutions. Regular security assessments ensure ongoing evaluation and reinforcement of defense mechanisms. Similarly, establishing robust monitoring systems tied with comprehensive incident response plans enables swift identification and mitigation of potential breaches.

#### Regular security assessments

Regular security assessments, including gamified simulations, are crucial for financial institutions to detect and mitigate cyber threats successfully. These assessments provide essential feedback loops, allowing institutions to defend against emerging threats and improve security. By simulating real-world attack scenarios and incorporating gamification elements, like scoring systems, organizations can engage security teams, identify weaknesses, and initiate a culture of continuous improvement in cybersecurity practices (Luh et al., 2020).

#### Monitoring and incident response plans

The platform of regular security assessments informs the development of monitoring and incident response plans to ensure organizations can detect and respond effectively to combat cybersecurity threats on time. Employing continuous system monitoring along with the utilization of threat intelligence efficiently identifies any suspicious and irregular activities or patterns of cyber threats, as proposed by Luh et al. (2020). In parallel, organizations should develop comprehensive incident response plans, incorporating scenario-based training (Angafor, Yevseyeva & He, 2020). These measures are essential to protecting effective detection and response to cyber incidents, thus enhancing institutions’ overall cybersecurity.

#### User training and awareness

User training and awareness strengthen monitoring and incident response plans by equipping individuals with the skills to recognize irregularities and report incidents perfectly. Chowdhury & Gkioulos (2023) developed a personalized learning theory-based model for cybersecurity training, utilizing game-based scenarios like Cyber CIEGE, enhancing bank employees’ skills and decision-making abilities against cyber threats. Chattopadhyay et al. (2023) emphasized the importance of cybersecurity in gaming, advocating for prevention strategies, user education, and collaboration between game developers and cybersecurity experts to enhance a safer gaming environment. “The Simulated Critical Infrastructure Protection Scenarios (SCIPS) gamified environment effectively raises awareness among senior executives about cyber-attacks on critical infrastructure, motivation discussions on cybersecurity investment prioritization, enhancing understanding of threats, and encouraging proactive measures (Cook et al., 2016). These initiatives emphasize the significance of proactive training, collaboration, and awareness-building in mitigating cyber threats across various sectors. Prioritize user awareness training through interactive modules and educate employees about potential cyber threats as well as how to identify them (Chowdhury & Gkioulos, 2023; Kävrestad et al., 2022).

#### Phishing detection

User training and awareness play a critical role in preventing and detecting phishing attacks, as educated users are better equipped to recognize phishing attempts, understand the risks involved, and respond appropriately, thereby reducing the impact of such attacks. Chowdhury & Gkioulos (2023) developed a personalized learning theory-based model based on cybersecurity training that integrates and incorporates game-based situations like Cyber CIEGE, offering an engaging platform for bank employees to develop their cybersecurity skills and emphasizing cybersecurity’s significance in gaming platforms, highlighting serious games like Anti-Phishing Phil for user training. Similarly, Hale, Gamble & Gamble (2015) presented CyberPhishing, a game-based platform on the most trending phishing awareness testing, demonstrating its effectiveness through three testing phases to refine and implement game-based phishing training strategies. These initiatives highlight the importance of gamification in detecting and mitigating phishing attacks within financial institutions, emphasizing the need for innovative approaches to cybersecurity training in simulated environments. These detection measures aim to proactively identify and mitigate game-based cyber threats within financial institutions, ensuring the security of sensitive data and operations. Table 9 explains the research sources, focuses on prevention/detection, and delineates game-based parameters, research objectives, key findings, and limitations, offering a holistic perspective on cybersecurity and gamification studies.

**Table 9:** Cyber-threats prevention and detection.

Reference	Prevention/Detection	Game-based parameters	Purpose of research	Key findings	Limitation
Angafor, Yevseyeva & He (2020)	Incident response plan	Serious games (SGs) add an extra layer of interactivity and entertainment	Examines the increasing concern of cybersecurity breaches and the shortage of skilled Cyber Security Incident Response (CSIR) professionals.	Tabletop Exercises (TTXs) enhance CSIRT (Cyber Security Incident Response) training effectiveness	Highlights the dependence on assumptions during training
Luh et al. (2020)	Confidentiality, integrity, Authentication, and Remote Access controls	Gamification elements include taxonomies, security standards, and data mapping mechanisms to simulate cyber-attacks.	Creating a robust defense strategy in the gamified model by mapping primary control	Strategically linked controls provide systematic defense against cyber threats	Gamified model faces challenges: scalability and control accuracy
Hale, Gamble & Gamble (2015)	Thwart phishing attacks	A game-based learning environment termed CyberPhishing	Develop a game-based learning platform called CyberPhishing to educate users about phishing threats effectively	A game-based approach integrating realism and training features to enhance user awareness of phishing threats and outlining a methodology for experimentation	Ethical concerns, complexity of implementation, and real-world validation issues
Kävrestad et al. (2022)	Awareness and training Information Security Awareness Training (ISAT). Context-based micro-training (CBMT)	Platform of a game	Training methods aim to enhance user behavior and secure practices in identifying phishing emails	Prevention measures contribute to improving user behavior and accurately identifying phishing emails, emphasizing the role of training in cybersecurity awareness and prevention.	Potential biases due to participation in simulated experiments, challenges in replicating natural environments, and the study’s focus on a specific demographic (university participants).
Chowdhury & Gkioulos (2023)	Training by developing a personalized learning	Cyber CIEGE game environment	Two cybersecurity training exercises were created using the model: game-based scenarios with Cyber CIEGE and a table-top team exercise.	The game-based tool, Cyber CIEGE, was engaging and useful for learning basic cybersecurity concepts.	Small sample size, participant feedback without objective performance metrics, Absence of a direct comparison with traditional training methods
Chattopadhyay et al. (2023)	Cybersecurity awareness	Game level design, player interaction, scoring systems, and game objectives	Promote essential awareness and offer guidance to support users and developers in online gaming.	Users express concerns about the security of their data during gaming, indicating a willingness to adopt improved cybersecurity measures	Comprehensive solutions or strategies to mitigate the identified threats
Cook et al. (2016)	Awareness of cybersecurity issues	Investment options, decision points, scoring system	Demonstrate the strategic implications of cyber-attacks on critical national infrastructure facilities, specifically in the context of investment decision-making by senior executives	Highlighting the strategic impact of cyber-attacks on shareholder value and the effectiveness of gamification in raising awareness of cybersecurity issues	Assessing the effectiveness of gamification in changing perceptions and behaviors

**RQ4: How and which AI tools be strategically integrated to enhance cybersecurity measures and mitigate cyber threats within gaming platforms in the financial sector?**

Gamification significantly boosts AI tools for cybersecurity by integrating elements of game design into training, behavioral analysis, encouraging security practices, and incident response, thus enhancing their effectiveness (Zeadally et al., 2020). As machine learning is the subfield of AI and the intersection of machine learning and gamification, highlighting their potential cooperative relationship and varied application (Swacha & Gracel, 2023).

### AI tools for cybersecurity

In today’s digital landscape, merging gaming platforms with financial services requires robust cybersecurity measures. To explore the intersection of AI, machine learning, and gamification aims to highlight state-of-the-art approaches to mitigate cyber risks and maintain a robust security framework in the financial gaming sector. Drewniak & Posadzińska (2020) investigated the relationship between learning and development tools and innovation in the artificial intelligence sector.

#### Machine learning algorithms

The best AI tools for cybersecurity often depend on machine learning algorithms to improve threat detection, incident response, and overall security features. Machine learning algorithms are critical in strengthening cybersecurity measures from various perceptions, including gaming platforms in the financial sector. Zeadally et al. (2020) discussed integrating AI tools with ML techniques like naïve Bayes, which handles diverse attributes and data classification, while decision trees help in classification and outcome prediction phenomena. Support vector machines (SVMs) enhance security through classification tasks, and k-nearest neighbor (k-NN) facilitates data classification and anomaly detection. Moreover, artificial neural networks (ANNs) enable complex pattern recognition parameters, and self-organizing maps (SOMs) are applied to cluster and visualize cybersecurity data. Deep neural networks (DNNs) detect offensive language and behavior in gaming platforms, and generative adversarial networks (GANs) generate synthetic data to strengthen cybersecurity defenses. These algorithms collectively contribute to the robustness of cybersecurity measures, enabling effective threat detection and response in gamification environments within the financial sector.

#### Reinforcement learning

In AI, machine learning algorithms and reinforcement learning are closely related to this versatile platform. Tao, Akhtar & Jiayuan (2021) discussed that AI tools greatly assist in applying game theory to cybersecurity by analyzing complex decision-making scenarios and powering the process for defenders in security games. Reinforcement learning algorithms, particularly Q-learning—where agents learn optimal actions by maximizing rewards through trial and error—play a crucial role in enhancing cybersecurity by allowing defenders to learn the best strategies within security games, even with limited or little knowledge about adversaries’ intentions. As highlighted by Tao, these AI tools automate decision-making processes, optimize strategies, and enhance compliance with evolving threats by analyzing vast amounts of data to predict adversaries’ behavior. Further, Zysman, Nitzberg & On (2020) shed light on the context of AI governance and game theory, where the strategic deployment of AI tools, particularly reinforcement learning algorithms, is crucial for understanding decision-making dynamics, enhancing security, and facilitating informed decision-making in governance and regulatory compliance in game theory contexts. This empowers defenders to respond proactively to cyber threats, ultimately enhancing cybersecurity defense effectiveness and safeguarding against anomalies.

#### Natural language processing

AI also plays a crucial role in natural language processing (NLP), closely associated with ML. ML algorithms, RL, and NLP are interconnected fields that often collaborate to develop advanced AI systems capable of understanding, generating, and interacting with human language. Rawindaran, Jayal & Prakash (2021) contributed to the field of NLP gamification platforms by highlighting the importance of natural language processing algorithms in enhancing fraud detection capabilities within gaming platforms in the financial sector. By analyzing user interactions, NLP algorithms can enable real-time threat response and mitigation strategies, thus safeguarding sensitive data and transactions against emerging cyber threats. This integration of AI-driven solutions, like NLP, reinforces the effectiveness of gamification platforms in detecting and preventing fraud, ultimately ensuring comprehensive protection of financial assets and customer information in the dynamic paradigm of online gaming and financial transactions. Employed to analyze user interactions within gaming platforms and enhance fraud detection capabilities to avoid anomalies.

#### Adversarial sandbox adaptive serious game approach

The adversarial Sandbox Adaptive Serious Game approach integrates the features of ML, RL, and NLP techniques into an interconnected cybersecurity training and defense framework. Integrates AI-driven non-player character (NPC) interactivity based on player responses to enhance cybersecurity education and skill development for better results. The study by Mittal et al. (2021) contributes to adversarial sandbox environments by proposing integrating AI tools within a game-based learning environment for cybersecurity education in blockchain technology to make networks efficient and secure. By employing an adversarial sandbox adaptive serious game approach coupled with AI-driven NPC interactivity based on player responses, the study proposes to address barriers to adopting blockchain technology. The serious game facilitates cybersecurity skill development and knowledge acquisition by continuously adapting NPC behaviors and gameplay dynamics informed by player responses.

Moreover, integrating AI tools with gamification strategies offers a comprehensive solution for enhancing cybersecurity in the financial sector, enabling organizations to reinforce threat detection and incident response mechanisms. Collaborative efforts are essential to establish standardized cybersecurity protocols, fostering proactive measures against evolving cyber threats. Table 10 provides a comprehensive summary of the integration of AI tools into gamification strategies for mitigating cyber threats. It highlights the effectiveness of various AI tools, presents key findings from relevant studies, and identifies limitations associated with each approach in cybersecurity applications. This structured overview serves to illustrate how these tools contribute to enhancing security measures within gaming platforms in the financial sector while also acknowledging the challenges that need to be addressed for optimal implementation.

**Table 10:** Artificial intelligence tools integrated cyber security.

Reference	AI tool	How to mitigate the cyber threat	Purpose of research	Key findings	Limitation
Tao, Akhtar & Jiayuan (2021)	Q-learning Algorithm (reinforcement learning)	Enhance game theory by providing automated decision-making processes in security games,	AI can analyze large volumes of data to identify patterns and predict adversaries’ behavior.	AI tools help defenders stay ahead by using game theory to make decisions automatically, find the best strategies, and stay ahead.	Difficulty in modeling real-world cyber-attacks, predicting adversaries’ actions, potential biases in AI decision-making
Zysman, Nitzberg & On (2020)	Deep learning neural networks (reinforcement learning)	Stakeholders detect and respond to cyber threats in real-time to enhance security measures within a game-theoretic framework	Examine the role of artificial intelligence (AI) governance within a game-theoretic framework,	Using AI algorithms like reinforcement learning to govern AI technologies effectively, enhancing security and decision-making	Insufficient focus on implementation challenges and human factors impacting governance in cyber threats
Zeadally et al. (2020)	ML technique, Deep Neural Networks (DNNs) and Generative Adversarial Networks (GANs	Integration of AI tools and gamification mitigates cyber threats while fostering security awareness.	Explore the effectiveness of integrating AI tools with gamification in cybersecurity to engage professionals.	Enhance threat detection, streamline response processes, and cultivate a culture of security awareness.	Addressing ethical concerns and simulating cyber threats in gamification pose challenges.
Mittal et al. (2021)	Continuous Learning Algorithm for NPC	The adversarial sandbox adaptive serious game approach enhances cybersecurity competencies in blockchain technology.	Serious game approach with AI enhancements for blockchain training to overcome adoption barriers	Incorporating AI to enhance NPC interactivity and highlighting the potential for large-scale workforce development in blockchain and cybersecurity through gamification	Rising complexity in blockchain architecture hindering its adoption, the lack of serious games for blockchain training
Rawindaran, Jayal & Prakash (2021)	Machine learning algorithms, reinforcement	Strengthen cybersecurity *via* continuous monitoring and threat response	Investigate the adoption of machine learning cybersecurity (MLCS) techniques within small and medium enterprises	Propose AI integration in financial gaming for cybersecurity, policy, funding enhancement	Narrow scope, time sensitivity, and resource constraints
Drewniak & Posadzińska (2020)	Machine learning algorithms, reinforcement	Adaptive response mechanisms and predictive analytics to proactively prevent attacks	Investigate the relationship between the utilization of learning and development tools and the innovation potential of enterprises operating within the artificial intelligence sector.	Modern knowledge management tools positively impact innovativeness in the artificial intelligence sector, particularly among programmers.	Potential biases in self-reported survey data and a focus solely on enterprises in Poland, limiting generalizability

## Taxonomy of personalized and gamification-based cyber threats

**Figure 5:**
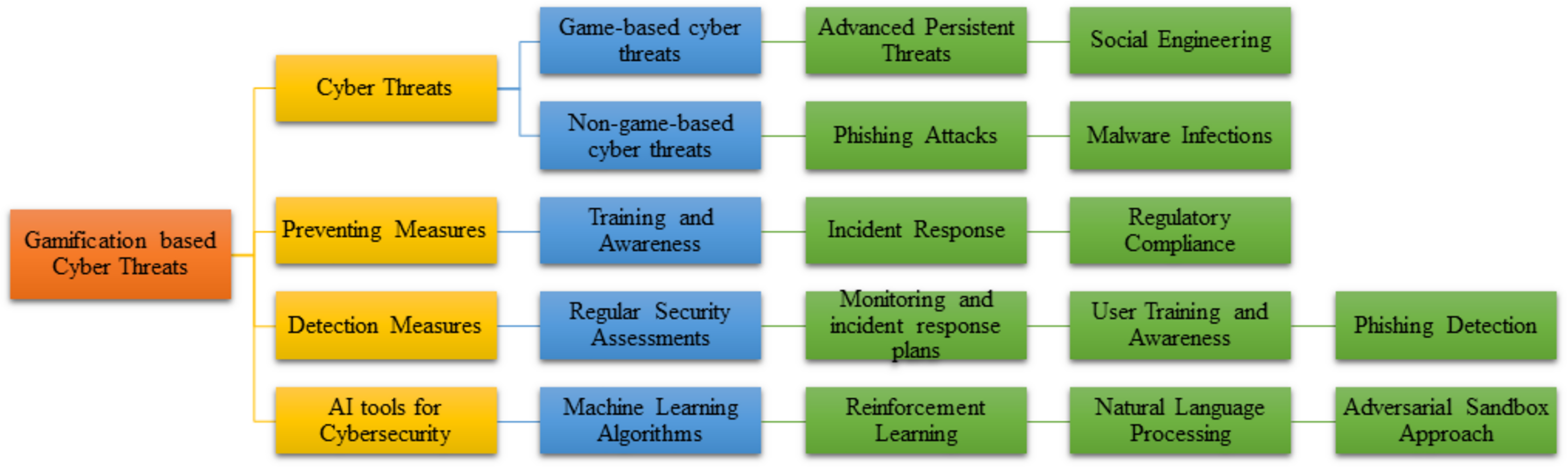
Taxonomy of personalized and gamification-based cyber threats.

Cybersecurity threats targeted at financial institutions and personalized activities have increasingly customized their malicious actions to specific users or organizations. Gamification strategies incorporate game-like elements to engage and educate users. This approach enhances user awareness and encourages proactive participation in cybersecurity practices. Financial institutions must understand and actively combat these threats to protect their assets, data, and reputation in an ever-evolving cyber landscape. To address this challenge, a taxonomy of personalized and gamified cyber threats in financial institutions is presented in Fig. 5. This taxonomy provides a structured framework for categorizing and analyzing the diverse threats directed at financial organizations. The taxonomy categorizes cyber threats into game-based and non-game-based, addressing prevention and detection measures using AI tools. Game-based threats encompass phishing attacks, malware infections, and advanced persistent threats, while non-game-based threats include social engineering and regulatory compliance issues. Prevention and detection measures involve regular security assessments, user training, and monitoring with incident response plans. AI tools like machine learning algorithms, specifically reinforcement learning and natural language processing, enhance detection capabilities, particularly in phishing detection, and improve incident response through adversarial sandbox approaches.

## Limitations

As the financial sector embraces game-based cybersecurity strategies, the potential for growth and innovation is clear. However, limitations in personalized gamification remain, such as difficulties in keeping content current, preventing user disengagement, and measuring effectiveness. Small and medium-sized enterprises (SMEs), usually defined by their limited workforce and budget, face resource constraints, and the added complexities of regulatory compliance and confidentiality protocols make it even more challenging to integrate and monitor gamified systems within the industry. Game-based cybersecurity awareness faces several challenges and limitations. The constantly evolving nature of cyber threats makes it difficult to keep game content current, which can reduce its effectiveness. Moreover, users may experience ‘security fatigue,’ where repeated exposure to game simulations leads to disengagement, diminishing the program’s impact. Measuring the effectiveness of game-based training is also challenging, as measuring human behavior and learning outcomes is less straightforward than evaluating technological solutions. Also, resource constraints, especially for SMEs, limit their ability to invest in high-quality game-based programs, reducing their overall effectiveness in enhancing cybersecurity awareness (Sharma & Thapa, 2023). Implementing gamification strategies in the financial sector faces significant challenges concerning regulatory compliance and confidentiality protocols. The industry’s severe regulatory framework makes it difficult to integrate gamified systems without breaching data privacy laws or security regulations. Confidentiality protocols further impede the ability to track and measure employee behavior in gamified training environments, limiting insights into the program’s effectiveness. Moreover, maintaining a balance between gamified engagement and adherence to strict compliance rules can reduce the overall impact of cybersecurity training programs. Finally, the cost and complexity of implementing gamification, including adhering to regulatory standards, present additional barriers for financial institutions (Bitrián et al., 2024).

## Future direction

As the financial sector increasingly adopts game-based cybersecurity strategies, it is ready for better growth and innovation. Looking ahead, the future direction of gamification in cybersecurity holds immense promise for enhancing threat detection, response mechanisms, and user engagement within financial institutions. Hence, the convergence of machine learning and gamification highlights their transformative impact on user experiences in information systems. It identifies various themes, like using gamification in machine learning education and machine learning in gamification research. Various methodologies and strategic understandings suggest promising ways for further exploration, emphasizing the effectiveness of combining machine learning and gamification. Despite limitations in data selection and publication bias, the study also contributes to advancing understanding in this under-researched area, providing a foundation for future investigation (Rawindaran, Jayal & Prakash, 2021; Swacha & Gracel, 2023). Future research in e-banking software design should focus on exploring the interconnection of user perceptions, considering factors like country, culture, moderator, and user profiles. There is an urgent need to address an additional practical study to validate and expand proposed categories and dimensions of user perceptions, aiming to provide a deeper understanding of user experiences for presenting better results. Creative efforts are necessary to identify new elements and functionalities to improve gamified business software development, focusing on different business contexts and user demographics. Further investigation should also aim to uncover correlations between design categories or dimensions along with user experiences, as well as explore similarities with other types of products or services (Rodrigues, Costa & Oliveira, 2016).

In finance, gamification offers a promising platform to strengthen security measures by engaging users in interactive and educational experiences. Financial institutions can increase user awareness of security protocols by integrating gamified elements, like simulations and challenges. Measuring the impact of gamification on financial security includes assessing changes in user behavior, risk mitigation, and incident response effectiveness. Integration with broader management practices ensures alignment with organizational goals and strategies, promoting a holistic approach to security enhancement. Ultimately, incorporating gamification in the finance sector also holds the potential to create a more resilient and proactive security environment against emerging cyber threats (Wanick & Bui, 2019). Creating gamified applications for accountability requires designing interactive platforms promoting transparency and responsibility. These applications should flawlessly integrate with various management domains, fostering collaboration and consistency. Additionally, addressing cultural variations ensures the effectiveness and relevance of accountability measures across different organizational contexts. Ultimately, such gamified solutions promote a culture of accountability while aligning with broader management objectives (Wanick & Bui, 2019). Integrating AI and ML technologies will continue to assist banks in various aspects of innovation and product development within the financial sector. Firstly, advancements in AI and ML algorithms will enable banks to enhance their predictive analytics capabilities further, allowing for more accurate prediction of market trends and consumer behaviors (Onari et al., 2024). Additionally, improving AI-powered recommendation systems will enable banks to deliver even more personalized product offerings, enhancing customer satisfaction and loyalty perspectives. Moreover, as AI and ML technologies become more sophisticated, banks will be better equipped to assess and mitigate risks associated with new product development initiatives, leading to more informed decision-making and resource allocation. Overall, the continued integration of AI and ML technologies will empower banks to stay ahead of the curve, drive innovation, and maintain competitiveness in the dynamic banking landscape of the future (Kurmanova, Kurmanova & Nurdavliatova, 2020). Ultimately, this research can contribute to a comprehensive understanding of design requirements and user experiences across various contexts and user groups.

## Conclusion

The review highlights the importance of incorporating gamification strategies to respond against the evolving cyber threats within the financial sector. Drawing insights from valued academic sources from 2015 to 2024, this study outlines the diverse threat landscape financial institutions face, ranging from conventional attacks to sophisticated, persistent threats. Moreover, the review identifies key methodologies for detecting and preventing emerging threats within financial gaming environments through a particular analysis of game-based and non-game-based cyber threat platforms. Additionally, the inquiry underscores the critical importance of AI tools in strengthening cybersecurity protocols within gaming environments, providing valuable strategic insights to strengthen overall cyber defense. This categorization into a taxonomy offers a smart way to clarify the various aspects of this augmentation. Future research should prioritize the development of personalized gamification strategies within the financial sector, focusing on the adaptation of these approaches to diverse user demographics and business contexts. Additionally, it is crucial to explore how AI-driven gamification can be harnessed to enhance cybersecurity measures, thereby strengthening defenses against the increasingly sophisticated threats of the digital age.

## Additional Information and Declarations

### Data Availability

The following information was supplied regarding data availability:

This study is a literature review.
